# Recurrent Hyperparathyroidism Due to Hyperactive Ectopic Intrathymic Parathyroid Gland 20 Years After Parathyroidectomy

**DOI:** 10.7759/cureus.107144

**Published:** 2026-04-16

**Authors:** Enas F Younis, Roleen R Toma, Aws A Ghraiz, Aseel Q Al-Omari, Abdalla M Alkhashmani

**Affiliations:** 1 Internal Medicine/Endocrinology, Istishari Hospital, Amman, JOR; 2 Internal Medicine, Istishari Hospital, Amman, JOR; 3 Internal Medicine, Hashemite University, Zarqa, JOR; 4 Pathology and Laboratory Medicine, Istishari Hospital, Amman, JOR; 5 Radiology, Istishari Hospital, Amman, JOR

**Keywords:** brown tumor, ectopic parathyroid adenoma, parathyroid disorder, recurrent parathyroid adenoma, surgical retreatment

## Abstract

Primary hyperparathyroidism is most commonly caused by a single adenoma, with multiglandular disease accounting for a smaller proportion of cases.

A recurrent hyperparathyroidism decades after prior parathyroidectomy is uncommon and often presents with nonspecific multisystem symptoms that delay diagnosis. Ectopic parathyroid tissue, particularly in intrathymic or retrosternal locations, represents a challenging but important cause of persistent or recurrent disease. A 43‑year‑old woman with a history of three‑gland parathyroidectomy 20 years earlier presented with a year of progressive fatigue, generalized weakness, recurrent colicky abdominal pain, persistent nausea, and an 8‑kg unintentional weight loss. She also reported diffuse arthralgia, proximal myalgia, and significant neuropsychiatric symptoms, prompting psychiatric evaluation. Laboratory studies revealed hypercalcemia (10.9 mg/dl), markedly elevated parathyroid hormone (PTH) (1235 pg/ml), hypophosphatemia, vitamin D insufficiency, and hypomagnesemia.

Imaging demonstrated destructive maxillofacial lesions, multiple sclerotic skeletal lesions, and persistent technetium‑99m sestamibi uptake in the mediastinum, consistent with an ectopic retrosternal parathyroid gland. The patient underwent thoracic surgical excision of the mass, with intraoperative PTH drop from 1235 pg/ml to 371 pg/ml. Histopathology confirmed an intrathymic parathyroid gland with hyperplasia and a dominant oxyphil pseudo‑adenomatous nodule.

Following surgical excision, the patient experienced rapid biochemical normalization and complete resolution of her gastrointestinal, musculoskeletal, and neuropsychiatric symptoms. Serial outpatient follow‑up demonstrated the sustained normalization of calcium and PTH levels. This case highlights the importance of considering an ectopic intrathymic parathyroid tissue as a cause of late recurrent hyperparathyroidism, even decades after prior surgery. Persistent multisystem symptoms in patients with a history of parathyroidectomy warrant thorough biochemical evaluation and advanced imaging to ensure timely localization and definitive management.

## Introduction

Persistent elevation of parathyroid hormone (PTH) levels with hypercalcemia following parathyroidectomy, whether occurring in the immediate postoperative period or developing years later, represents a challenging clinical scenario that often indicates the presence of unrecognized or ectopic parathyroid tissue [1]. Accurate localization of abnormal parathyroid glands is therefore essential, particularly in cases of ectopic adenomas [2], which may escape detection during initial surgical exploration. Such ectopic glands are most commonly found in the mediastinum, where their atypical location complicates both diagnosis and surgical management [3].

Despite advances in preoperative imaging, including technetium‑99m sestamibi scintigraphy, magnetic resonance imaging (MRI), and positron emission tomography/computed tomography (PET/CT), localization remains difficult in reoperative cases [4]. The limitations of conventional imaging techniques contribute to the lower success rates observed in revision surgeries compared with primary procedures [5]. Consequently, a comprehensive, multimodal imaging approach is often required to improve diagnostic accuracy, guide surgical planning, and reduce operative risk [5]. The combined use of multiple imaging modalities, such as technetium‑99m sestamibi scanning, ultrasonography, and MRI, has been shown to enhance localization, particularly when standard techniques yield inconclusive results [4,5]. Specifically, the integration of advanced imaging modalities, including technetium-99m sestamibi single photon emission computed tomography (SPECT)/CT and multiparametric MRI, can significantly improve the precise anatomical and functional localization of elusive parathyroid adenomas or hyperplastic glands in the reoperative setting [6]. This is particularly important, as accurate preoperative localization strongly predicts operative success, whereas discordant or negative imaging findings are associated with an increased risk of surgical failure [6].

In this context, persistent hyperparathyroidism following parathyroidectomy warrants thorough evaluation to avoid delayed diagnosis, recurrent complications, and unnecessary surgical exploration [2]. This case report underscores the importance of meticulous preoperative assessment and highlights the diagnostic challenges associated with ectopic parathyroid adenomas.

## Case presentation

A 43-year-old woman presented with a one-year history of progressive fatigue, generalized weakness, and recurrent episodes of colicky abdominal pain lasting approximately 15 minutes each. She also reported persistent nausea and an 8-kg unintentional weight loss. Musculoskeletal symptoms included diffuse arthralgias and proximal myalgias. Neuropsychiatric manifestations, namely, low mood, anxiety, and sleep disturbances, prompted psychiatric consultation during hospitalization.

Her medical history was notable for a three-gland parathyroidectomy performed 20 years earlier for primary hyperparathyroidism. She was not on calcium or vitamin D supplementation.

On examination, she appeared fatigued but not in acute distress. Musculoskeletal examination revealed generalized tenderness. On examination, a well‑defined, firm swelling was noted over the right midface, extending from the infraorbital margin to the upper lip region. The overlying skin was intact, with normal color and temperature, and no regional lymphadenopathy was detected. The swelling produced noticeable facial fullness and mild obliteration of the right nasolabial fold. Abdominal and neurological examinations were unremarkable.

The results of the laboratory evaluation are shown in Table 1.

**Table 1:** Baseline biochemical evaluation at presentation.

Parameter	Result	Reference range
Total calcium (mg/dl)	10.9	8.6-10.0
Intact parathyroid hormone (pg/ml)	1235	15-65
Phosphorus (mg/dl)	1.4	2.5-5
25‑Hydroxyvitamin D (ng/ml)	20	30-70
Magnesium (mg/dl)	1.4	1.5-2.5

Radiological findings

**Figure 1:**
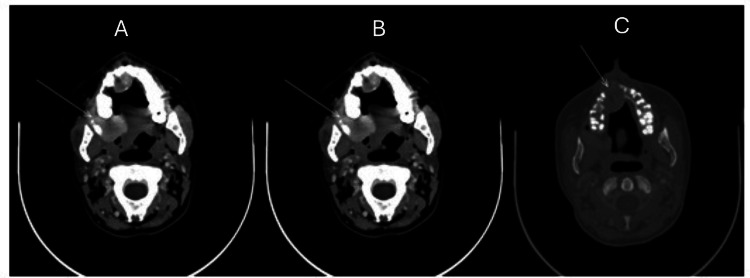
(A-C) Contrast‑enhanced CT of the maxilla showing a 2.6 × 2.2 × 2.1 cm expansile, enhancing destructive lesion (arrows) of the anterior upper jaw with extension through the hard palate into the oral cavity and associated cortical bone destruction. CT: computed tomography

**Figure 2:**
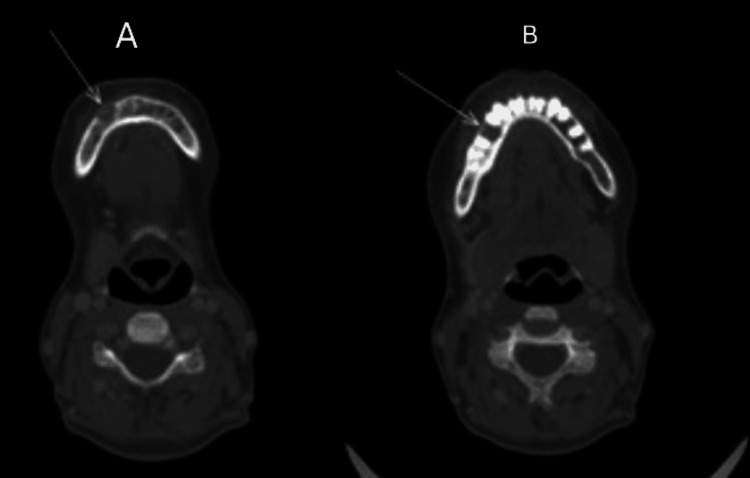
(A and B) Contrast‑enhanced CT of the right oral cavity demonstrating a 3.6 × 3.2 × 2.8 cm enhancing mass (arrows) with internal calcifications and soft‑tissue extension through the interval between the maxilla and mandibular ramus into the buccal soft tissues. CT: computed tomography

Contrast‑enhanced CT of the maxilla (Figure 1) and the oral cavity (Figure 2) revealed two enhancing destructive lesions involving the upper jaw and oral cavity.

**Figure 3:**
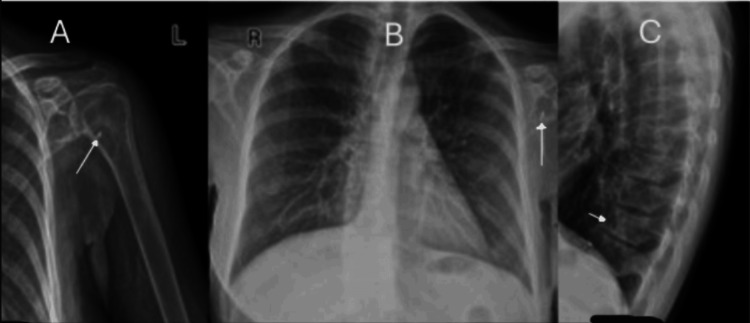
Skeletal survey radiographs showing sclerotic lesions (arrows) in the left humeral head (A), left scapula (B), and thoracic spine (T10-T12) and L1 vertebral body (C), consistent with metabolic bone disease in severe hyperparathyroidism.

**Figure 4:**
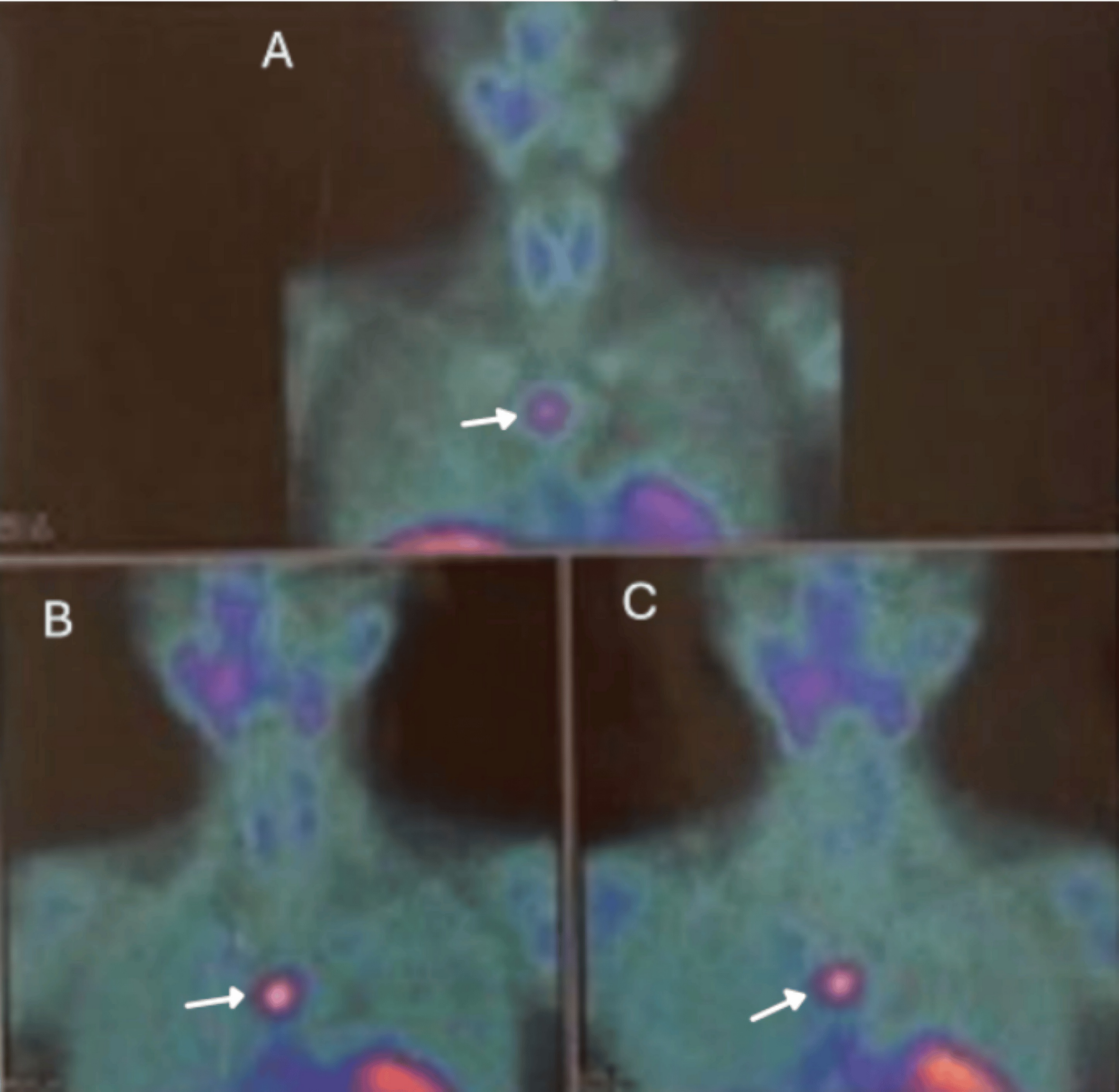
Technetium-99m sestamibi imaging demonstrating persistent retrosternal mediastinal tracer uptake (arrows); activity increases at 20 minutes (B) with absent washout on delayed images (C), localizing an ectopic parathyroid focus. (A) early image, (B) image at 20 minutes, and (C) delayed image.

Skeletal survey showed sclerotic lesions in the left humeral head, left scapula, thoracic spine (T10-T12), and L1 vertebra (Figure 3). Technetium-99m sestamibi scan (Figure 4) demonstrated persistent retrosternal tracer uptake without washout, consistent with an ectopic parathyroid gland.

Operative findings

**Figure 5:**
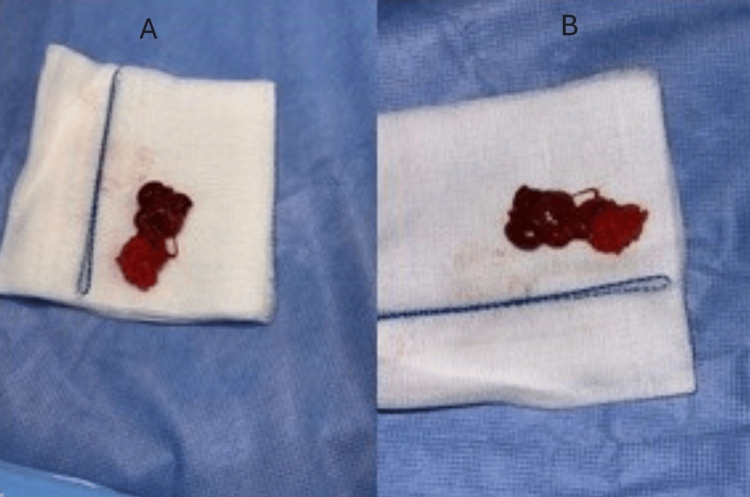
Intraoperative thoracic view showing the en‑bloc excision of a well‑circumscribed retrosternal mass (A and B) adherent to posterior mediastinal structures using monopolar energy; specimen consistent with ectopic intrathymic parathyroid tissue.

The patient subsequently underwent thoracic surgical excision of the retrosternal mass through a coordinated endocrine-thoracic approach. Intraoperatively, the lesion was identified as a well‑circumscribed mass adherent to the posterior mediastinal structures (Figure 5). Dissection was done using monopolar energy, and the mass was excised en bloc. Frozen‑section analysis confirmed parathyroid tissue, consistent with an ectopic hyperfunctioning gland. A marked biochemical response was observed during the procedure, with PTH levels decreasing from 1235 pg/ml pre‑excision to 371 pg/ml post‑excision, indicating the successful removal of the hypersecreting source. There were no immediate postoperative complications.

Pathological findings

Intraoperative consultation of the retrosternal nodule was requested from the histopathology department. The fresh specimen received was a lobulated, well-circumscribed soft brown mahogany nodule that measured 2 × 1.0 × 0.5 cm and weighed 3000 milligrams. An attached lobule of soft yellow fatty tissue, measuring 0.7 × 0.5 cm, was noted. Parathyroid gland tissue was confirmed on frozen section, and adherent thymic tissue was identified.

**Figure 6:**
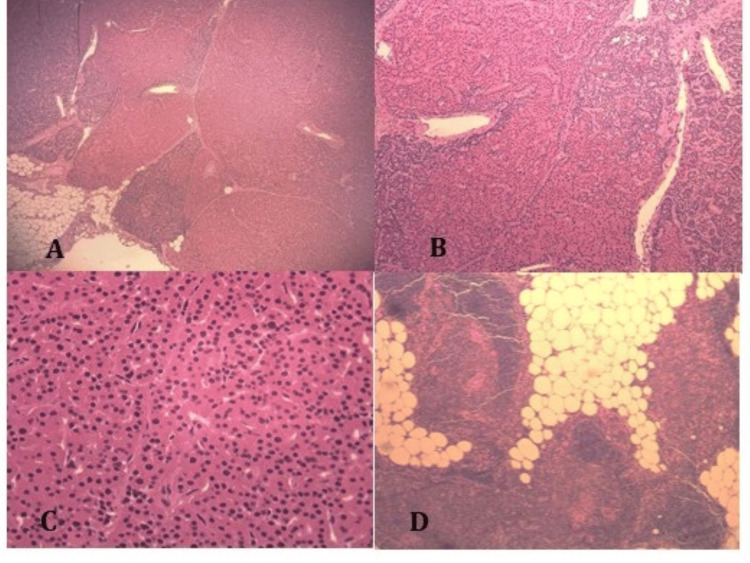
Histopathology of the ectopic parathyroid tissue revealed an enlarged and lobulated cellular parathyroid gland (A, H&E: 40×), with markedly reduced stromal fat, composed mainly of chief and oxyphil cells (B, H&E: 100×). A dominant pseudo-adenomatous nodule of oxyphils is present (C, H&E: 400×). Adjacent fatty tissue revealed non-neoplastic thymic tissue (D, H&E: 100×). H&E: hematoxylin and eosin

Permanent sections revealed a lobulated parathyroid gland tissue (Figure 6A) featuring diffuse hyperplasia of chief cells, oxyphil cells, and occasional clear cells, admixed with minimal fat (Figure 6B). No rim of compressed normal parathyroid gland tissue was identified. A distinct oval micronodule, surrounded by a thin fibrous pseudocapsule, was present, measured 8 mm, and predominantly (95%) consisted of oxyphil cells (Figure 6C). Sections from the adherent fatty tissue revealed non-neoplastic thymic tissue (Figure 6D). No evidence of atypical features in the form of fibrosis or hemosiderin is noted. No evidence of capsular or vascular invasion, necrosis, or infiltration into surrounding thymic tissue is seen. No evidence of malignancy is detected.

The final histopathology diagnosis was "ectopic retrosternal (intrathymic) parathyroid gland, with parathyroid hyperplasia and dominant oxyphil pseudo-adenoma".

Follow-up

Postoperatively, the patient was monitored in a high‑dependency setting with serial assessments of serum calcium, phosphate, and PTH levels. Within the first 24 hours, the patient developed a progressive decline in serum calcium, consistent with hungry bone syndrome, given the long‑standing hyperparathyroidism state and extensive skeletal involvement. The patient reported perioral tingling and mild distal paresthesia, which correlated with the biochemical drop.

Calcium replacement was initiated promptly using intravenous calcium gluconate, followed by transition to high‑dose oral calcium supplementation once symptoms stabilized. Active vitamin D analogs and oral magnesium were introduced to support intestinal calcium absorption and mitigate the severity of hypocalcemia. Electrolytes were monitored closely, with particular attention to magnesium and phosphate levels, and adjustments were made as needed (Table 2).

**Table 2:** Postoperative electrolyte trends and corresponding clinical interventions. Calcium replacement included IV calcium gluconate followed by high‑dose oral calcium plus calcitriol. ↓: decrease; ↑: increase; IV: intravenous

Post‑op day	Calcium	Phosphorus	Magnesium	Notes
0	↓	↓	—	Symptomatic hypocalcemia begins
1-2	↓↓	↓↓	↓	IV calcium initiated
3-5	Improving	Improving	Improving	Transition to oral calcium + calcitriol

Over the subsequent days, the patient's calcium levels gradually improved, and symptoms resolved. By the end of the first postoperative week, the patient was maintained on oral calcium and vitamin D with stable biochemical parameters.

**Figure 7:**
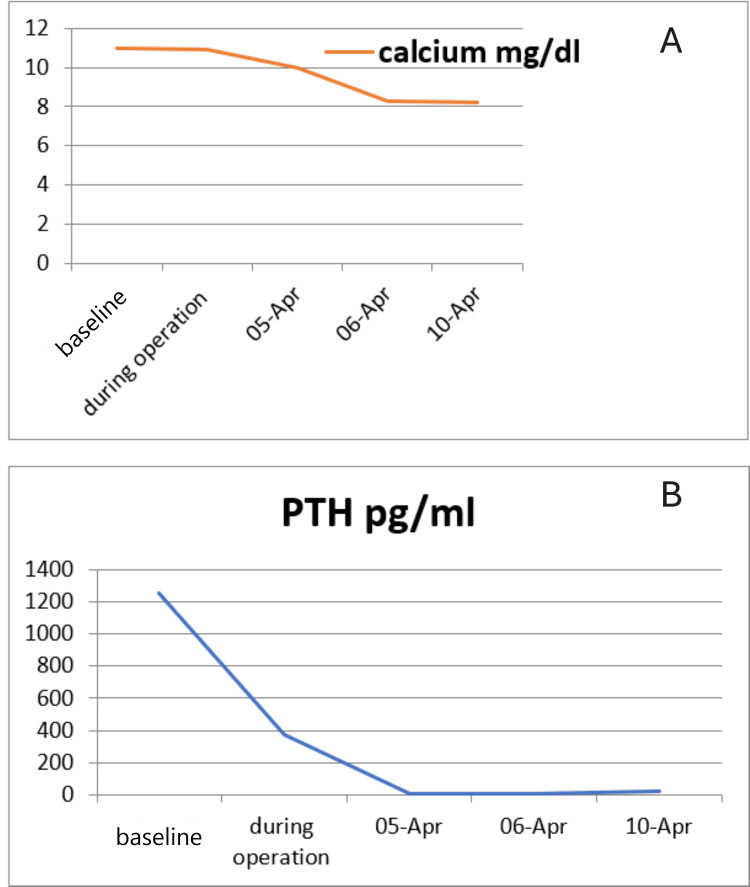
Changes in calcium (A) and PTH (B) levels before and after surgery. PTH level was 9, 6, and 23 pg/ml on the first, second, and fifth days post-op consecutively. PTH: parathyroid hormone

Serial outpatient evaluations postoperatively revealed the sustained normalization of calcium and PTH levels (Figure 7). The patient reported complete resolution of her previous symptoms, including fatigue, musculoskeletal pain, and gastrointestinal complaints. She expressed high satisfaction with her recovery. The facial swelling related to the maxillary brown tumor began to show early signs of regression, and the patient was scheduled for outpatient endocrine follow‑up and repeat imaging to document skeletal remodeling.

## Discussion

Brown tumors represent an advanced skeletal manifestation of uncontrolled hyperparathyroidism and occupy the severe end of the osteitis fibrosa cystica spectrum [7]. Although historically more prevalent, their incidence has diminished with the advent of routine biochemical screening and earlier diagnosis. These lesions arise from excessive osteoclastic activity, fibroblastic proliferation, and hemosiderin deposition, producing expansile osteolytic masses that may radiographically mimic primary bone neoplasms [7]. Maxillofacial involvement is particularly uncommon, making facial swelling an unusual initial presentation of hyperparathyroidism [8].

In this case, profoundly elevated PTH levels indicated long-standing disease consistent with extensive skeletal involvement. The identification of a retrosternal ectopic parathyroid adenoma highlights the necessity of considering ectopic tissue in patients with severe or persistent hyperparathyroidism [2,3]. Such adenomas, arising along the embryologic migration pathway, account for a notable minority of primary hyperparathyroidism cases and often require thoracic surgical access for definitive excision [9].

Intraoperative PTH monitoring provided the essential biochemical confirmation of successful gland removal, with the greater-than-50% decline within the expected 10-15-minute window validating adequate excision [10]. In this case, the decrease from 1235 pg/ml to 371 pg/ml corresponded with the frozen-section confirmation of parathyroid tissue.

The postoperative development of hypocalcemia was consistent with hungry bone syndrome, a predictable complication in patients with high bone turnover and extensive skeletal involvement [11]. Rapid remineralization following abrupt PTH reduction results in the significant redistribution of calcium, phosphate, and magnesium into bone. Management requires vigilant monitoring and aggressive supplementation with calcium and active vitamin D analogs to prevent symptomatic hypocalcemia and facilitate skeletal recovery [11].

Regression of brown tumors after the normalization of PTH levels is well documented, although the timeline varies depending on lesion size, chronicity, and patient age [12]. Many lesions regress spontaneously following metabolic correction, whereas others may require extended follow-up or, in rare cases, surgical intervention. The early improvement in maxillary swelling observed in this patient is consistent with the expected skeletal remodeling after successful endocrine control.

Overall, this case underscores several important clinical principles. Expansile maxillofacial bone lesions should prompt the consideration of metabolic bone disease, particularly when radiographic features deviate from those typical of odontogenic or primary bone tumors. Ectopic parathyroid adenomas remain a significant and sometimes overlooked cause of severe hyperparathyroidism, requiring systematic localization and multidisciplinary management [3]. Finally, proactive anticipation and management of postoperative calcium dynamics are essential, especially in patients with advanced skeletal involvement, to ensure safe recovery and optimize long-term outcomes.

## Conclusions

This case illustrates the diagnostic and therapeutic challenges associated with late recurrent hyperparathyroidism, particularly when originating from ectopic intrathymic parathyroid tissue. Although uncommon, ectopic glands remain an important cause of persistent or recurrent disease, even decades after initial parathyroidectomy. The patient's multisystem presentation, spanning gastrointestinal, musculoskeletal, neuropsychiatric, and maxillofacial manifestations, highlights the diverse and often nonspecific symptomatology that can obscure timely diagnosis. Advanced imaging played a critical role in localizing the ectopic retrosternal gland in this case, emphasizing the need for a multimodal approach when conventional studies are inconclusive. 

Successful surgical removal of the intrathymic gland resulted in the rapid biochemical normalization and progressive resolution of systemic and skeletal manifestations, including the regression of maxillofacial brown tumor-related swelling. This case reinforces the necessity of comprehensive evaluation in patients with a history of parathyroidectomy who present with persistent hypercalcemia or multisystem symptoms. Early recognition and targeted imaging are essential for accurate localization, effective surgical intervention, and the prevention of long-term complications.
